# Supplementary material for: Outer membrane protein assembly mediated by BAM-SurA complexes
Source: Nat Commun. 2024 Sep 1;15:7612. doi: 10.1038/s41467-024-51358-x (PMC11366764; doi:10.1038/s41467-024-51358-x)
Supplement: Supplementary file 1 — Supplementary Information [file 41467_2024_51358_MOESM1_ESM.pdf]

Outer membrane protein assembly mediated by BAM-SurA complexes

Katherine L Fenn[1]*, Jim E Horne[1,5]*, Joel A Crossley[1], Nils Böhringer[2,3,4], Romany J Horne[1,6], Till F. Schäberle[2,3,4], Antonio N Calabrese[1], Sheena E Radford[1†], Neil A Ranson[1†]

<u>Supplementary Information</u>

Supplementary Figure 1: Introductory Figure - Different conformations of BAM and SurA.
Supplementary Figure 2: Western blot and growth controls for complementation assays.
Supplementary Figure 3: Proteomics of three *E. coli* strains *ΔsurA*, *ΔsurA* complemented with wild-type *SurA* and *ΔsurA* complemented with *SurA(Δ23-28)*.
Supplementary Figure 4: CryoEM of of SurA:BAM disulphide linked via POTRA-1.
Supplementary Figure 5: CryoEM of SurA-cross-linked to POTRA-1 in the presence of darobactin-B.
Supplementary Figure 6: CryoEM of SurA-BAM complex cross-linked at POTRA-1 in the presence of WEYIPNV.
Supplementary Figure 7: CryoEM structure of SurA-OmpX:BAM cross-linked via POTRA-1.
Supplementary Figure 8: CryoEM structure of SurA_OmpX cross-linked to β1 of BamA.
Supplementary Figure 9: CryoEM structure of SurA_EspP:BAM cross-linked between β1 of BamA.
Supplementary Figure 10: Angular Distributions of final cryoEM maps.
Supplementary Table 1: CryoEM data collection parameters.
Supplementary Table 2: Modelling Statistics Table.
Supplementary Table 3: List of Plasmids.
Supplementary Table 7: List of Primers.
Supplementary Table 4: List of *E. coli* strains.
Supplementary Table 5: Single-molecule FRET PDA fitting results for SurA Core:PPIase1.
Supplementary Table 6: Single-molecule FRET PDA fitting results for SurA Core:PPIase2.

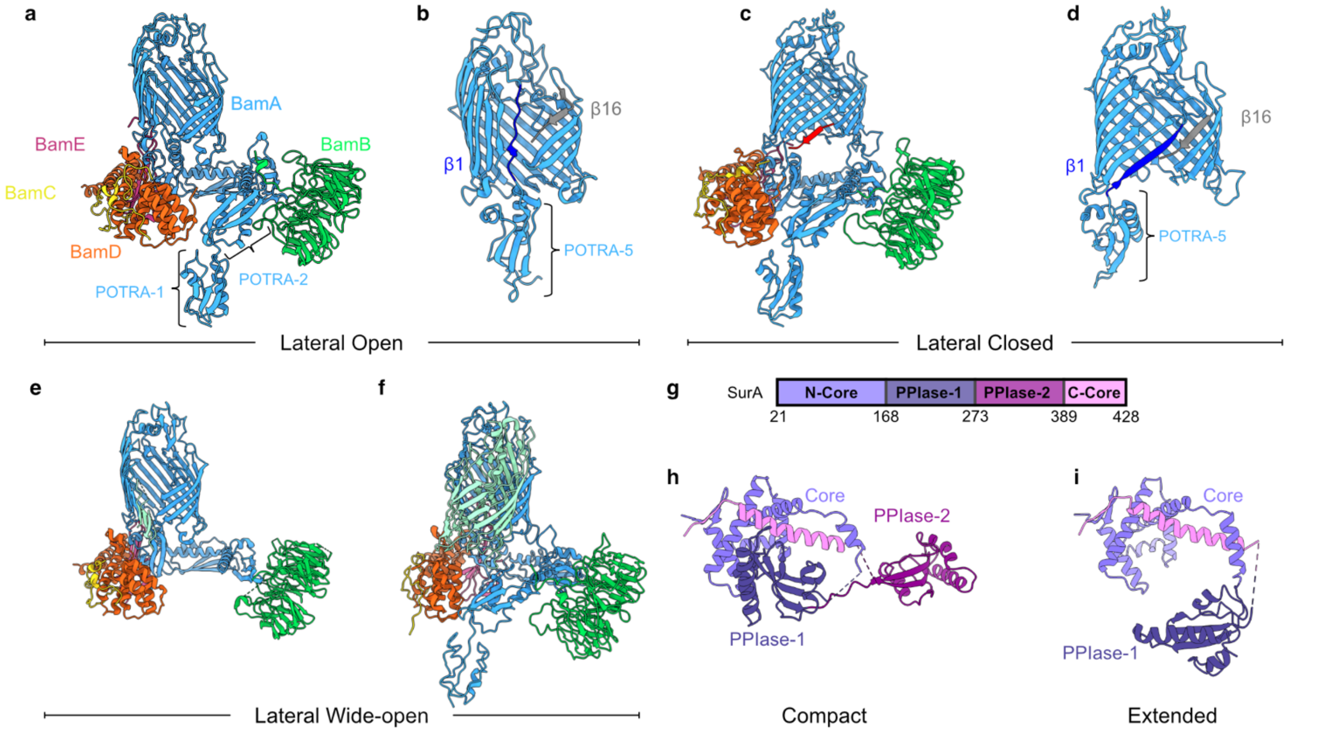

Supplementary Figure 1: Different conformations of BAM and SurA.

a. Structure of Lateral Open BAM (composed of five proteins, BamABCDE (the same colours are used throughout) (PDB = 5LJO)[1]. **b.** The Lateral Open BAM structure has β1 (navy) and β16 (grey) separated and POTRA-5 occluding access to the barrel lumen (BamA barrel and POTRA-5 shown only). **c.** Structure of Lateral Closed BAM (closed by binding darobactin-B (DAR-B), an antibiotic that mimics an OMP strand (coloured in red) (PDB = 8BVQ)[2]. **d.** Lateral Closed BAM has β1 (navy) and β16 (grey) hydrogen bonded and POTRA-5 has swung out revealing access to the barrel lumen (BamA barrel and POTRA-5 shown only). **e.** Structure of Wide Open BAM caught in the act of folding EspP (turquoise) where four strands of EspP are visible (PDB = 7TTC)[3]. Note POTRA-1, POTRA-2 and part of POTRA-3 were not resolved in this EM structure. **f.** Wide Open BAM structure where all 12 β-strands of EspP are visible (PDB = 7YE4)[4]. **g.** Schematic of the domain organisation of SurA (not to scale, these colours are used throughout). Note that residues are numbered from the N-terminus that contains the signal sequence (residues 1-20) for secretion into the periplasm. This numbering is used throughout. **h.** Structure of Compact SurA in which PPIase-1 is packaged against the Core domain (PDB = 1M5Y)[5]. **i.** Structure of Extended SurA in which PPIase-1 is dissociated from the Core domain (PDB = 2PV3)[6]. Note that PPIase-2 was not present in this construct. Dashed lines in **h** and **i** mark unmodelled residues.

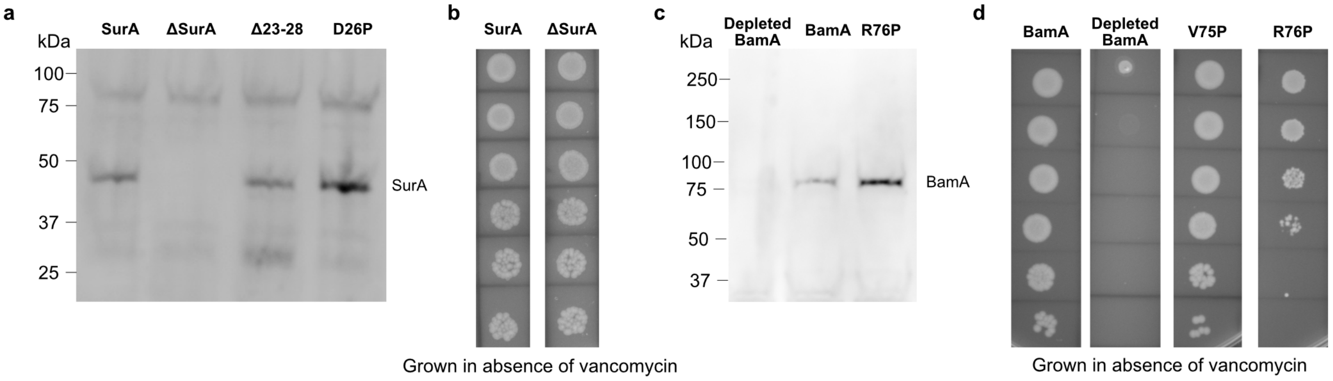

Supplementary Figure 2: Western blot and growth controls for complementation assays.
a. Western blot (using anti-SurA antibody) showing the expression levels of SurA in the ΔSurA bacterial strain and the ΔSurA strain complemented with plasmids expressing WT SurA, SurAΔ23-28 or SurA(D26P). **b.** Representative growths of bacteria (*ΔsurA*) complemented with plasmids expressing WT SurA or untransformed control (*ΔsurA*). Results are from n=3 biological repeats, one representative example is shown here. **c.** Western blot (using anti-His tag antibody) showing the expression levels of BamA in the depleted BamA bacterial strain and the strain complemented with plasmids expressing BamA, or BamA(R76P). **d.** Representative growths of the WT BamA, untransformed control (depleted BamA) and two proline variants, as indicated**.** Results are from n=3 biological repeats, with one representative example shown here. (Source data are provided in the Source Data file for a and c)

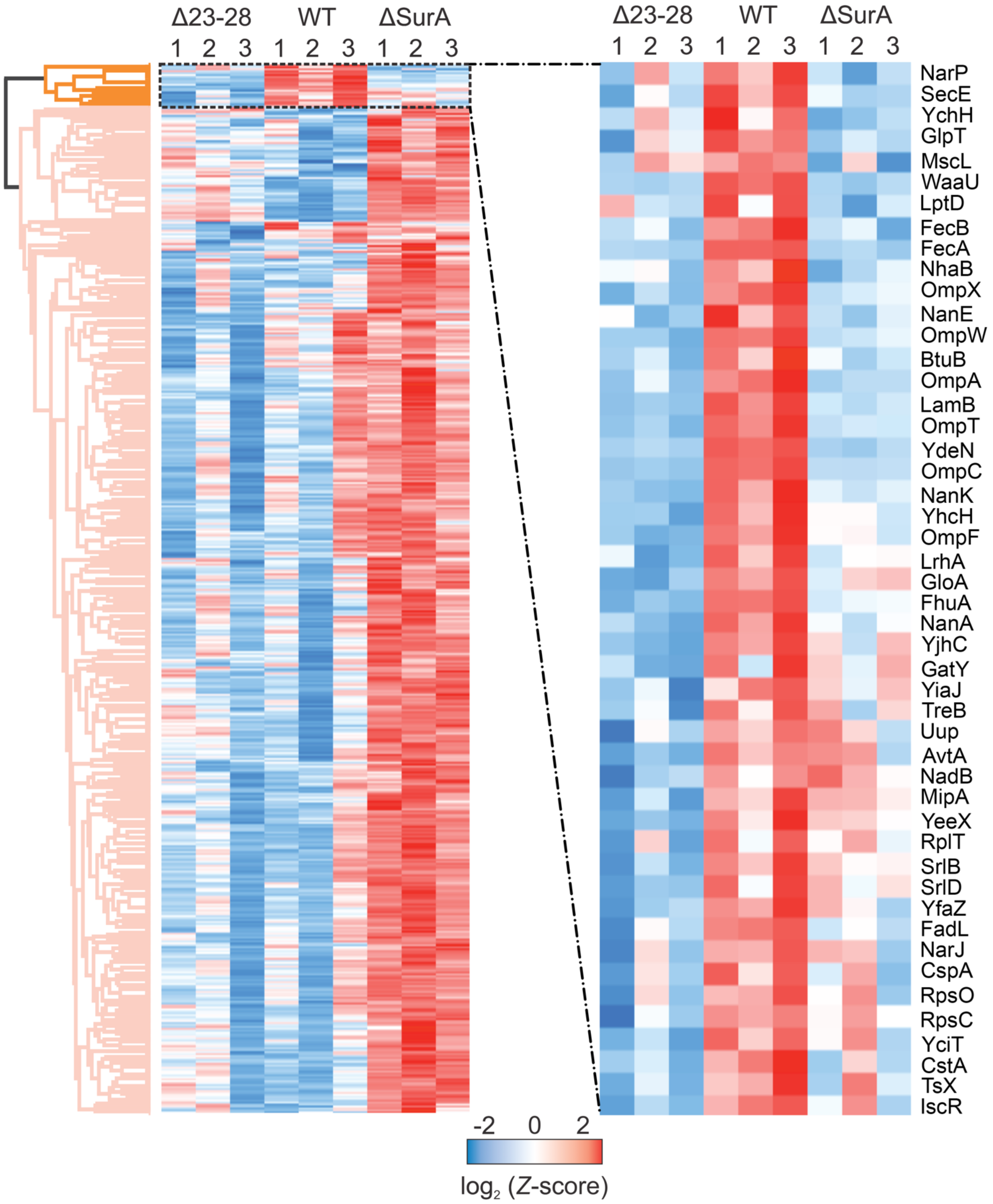

Supplementary Figure 3: Proteomics of three *E. coli* strains *ΔsurA*, *ΔsurA* complemented with wild-type *SurA* and *ΔsurA* complemented with *SurA(Δ23-28)*.

ANOVA was used to identify proteins that had a significant difference in abundance in one of the three samples (each sample has three biological repeats). Following ANOVA, hierarchical clustering was used to group proteins that have similar patterns in their changes in abundance. Many proteins were significantly upregulated upon SurA deletion (ΔSurA) (clusters highlighted on left in pale orange). A subset of proteins was downregulated (relative to protein levels in *ΔsurA* complemented with wild-type SurA) to similar levels in *both* the *ΔsurA* strain, and the *ΔsurA* strain complemented with *surA(Δ23-28)* (cluster highlighted in bold orange, and shown as a zoomed in view on the right-hand side). This includes many OMPs (**Fig. 2a**), as listed alongside, showing that residues 23-28 of SurA are critical for the folding of these proteins into the OM.

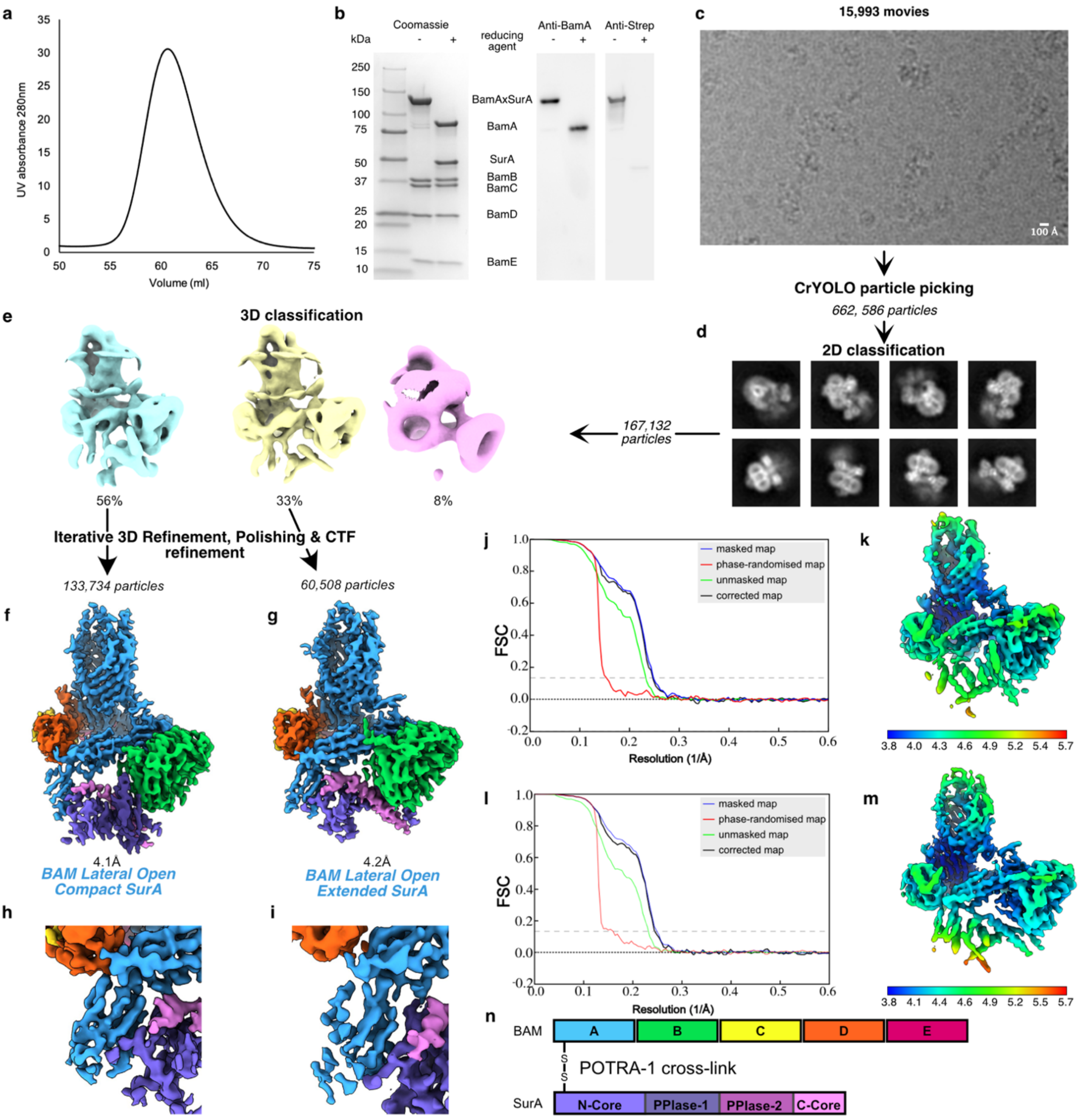

Supplementary Figure 4: CryoEM of of SurA:BAM disulphide linked via POTRA-1.
a. Gel filtration trace for purification of BamA(R76C)BCDE SurA(K27C). **b.** SDS PAGE gel and western blot of purified concentrated sample (SurA is Strep tagged). **c.** Representative raw electron micrograph and **d.** representative 2D classes. **e.** 3 class 3D classification with classes separated based on SurA conformation. Final reconstruction of **f.** BAM-Compact SurA and **g.** BAM-Extended SurA after iterative polishing/CTF refinement and 3D refinement coloured by protein subunit (BAM) and domain (SurA). Note that BAM is Lateral Open in these structures. **h.** POTRA-1 SurA N-terminus interface of Compact SurA. **i.** POTRA-1 SurA N-terminus interface of Extended SurA. **j.** FSC curve and **k.** structure coloured by local resolution of BAM-Compact SurA. **l.** FSC curve and **m.** structure coloured by local resolution for BAM-Extended SurA. **n.** Schematic showing the location of the introduced disulphide bond. (Source data are provided in the Source Data file for a and b)

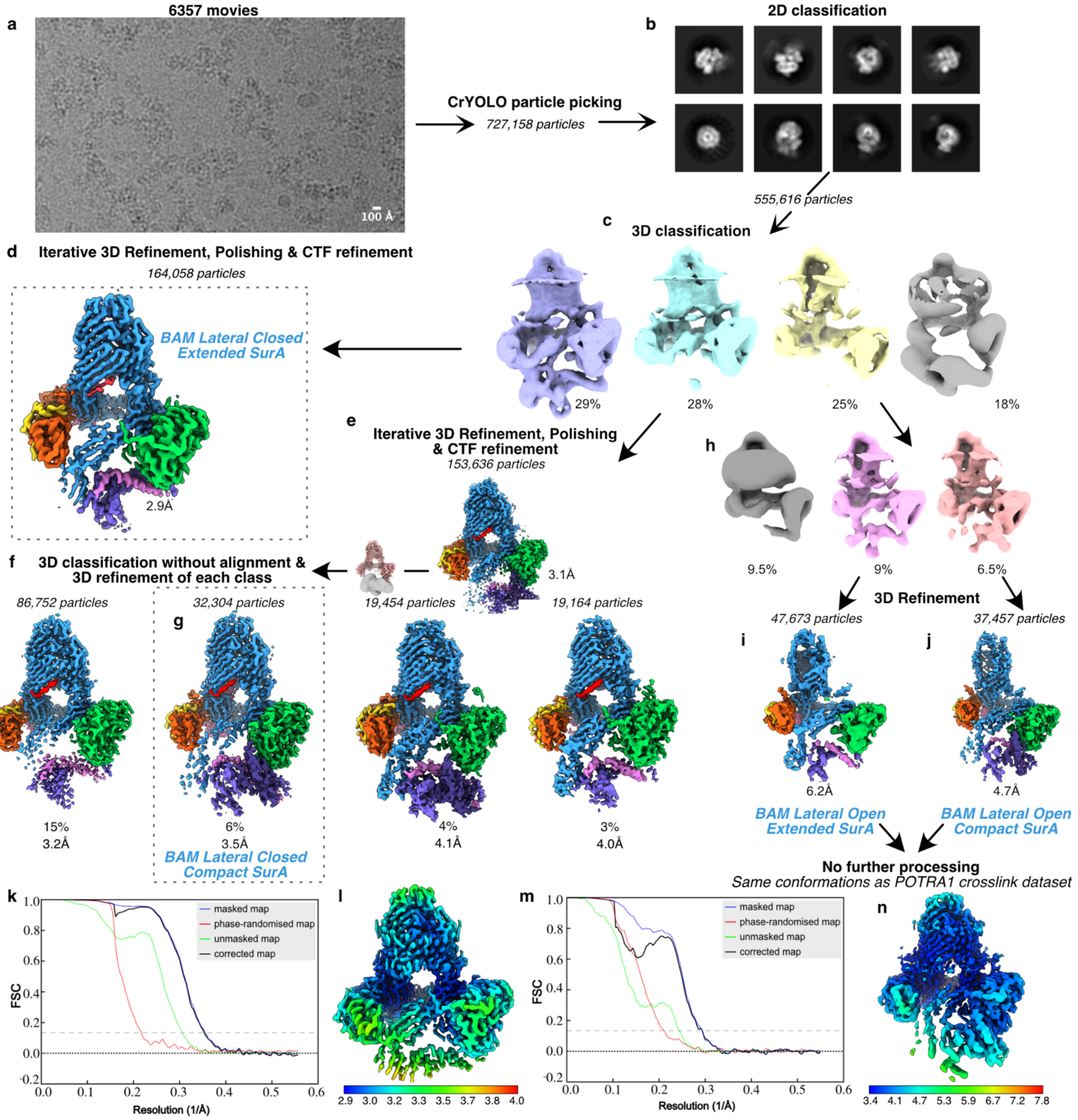

Supplementary Figure 5: CryoEM of SurA-cross-linked to POTRA-1 in the presence of darobactin-B.

a. Representative raw micrograph and **b.** representative 2D classes. **c.** 4 class 3D classification with 2 classes corresponding to Lateral Closed BAM, one class corresponding to Lateral Open BAM. **d.** Final reconstruction of BAM Lateral Closed Extended SurA after iterative polishing/CTF refinement and 3D refinement coloured by protein subunit (BAM) and domain (SurA). **e.** BAM Lateral Closed Compact SurA after iterative polishing/CTF refinement and 3D refinement coloured by protein subunit (BAM) and domain (SurA) showed poor resolution in POTRA-1 and SurA. **f.** 3D classification without alignment was used with the mask in grey to improve resolution of SurA and each class was 3D refined. **g.** Final Reconstruction of BAM Lateral Closed Compact SurA coloured by protein subunit (BAM) and domain (SurA). **h.** Lateral Open BAM was further classified and 3D refined into **i.** BAM Lateral Open Extended SurA and **j.** BAM Lateral Open Compact SurA. (**i and j**) are the same conformation as apo POTRA-1 dataset (**ED Fig. 2)**, no darobactin is observed and were not processed any further. **k.** FSC curve and **l.** coloured by local resolution for BAM Lateral Closed Extended SurA. **m.** FSC curve and **n.** coloured by local resolution for BAM Lateral Closed Compact SurA.

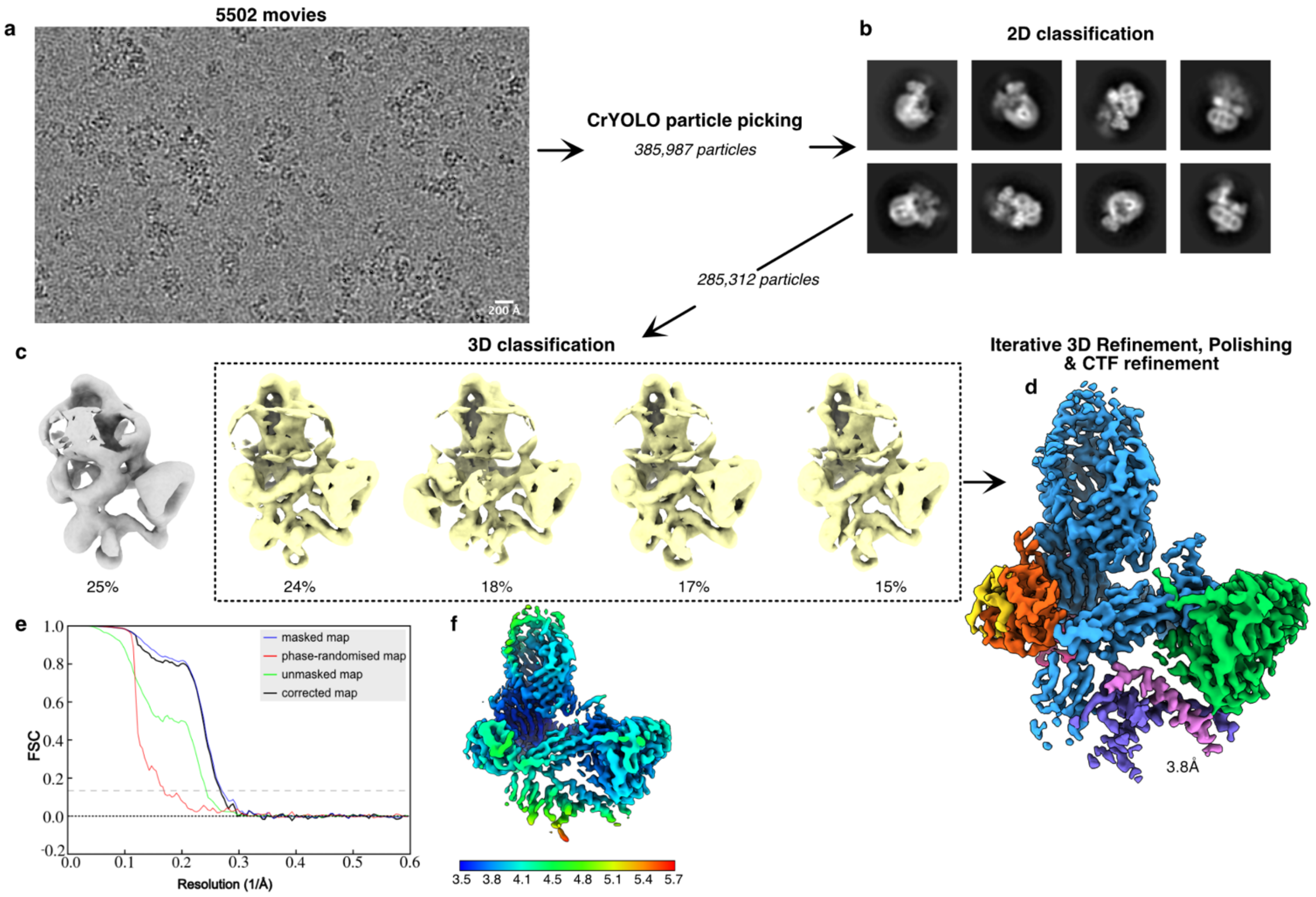

Supplementary Figure 6: CryoEM of SurA-BAM complex cross-linked at POTRA-1 in the presence of WEYIPNV.
a. Representative raw electron micrograph and **b.** representative 2D classes. **c.** 5 class 3D classification with one class of low-resolution particles and 4 classes combined for 3D refinement. **d.** Final reconstruction after iterative polishing/CTF refinement and 3D refinement coloured by protein subunit (BAM) and domain (SurA). **e.** FSC curve and **f.** final reconstruction coloured by local resolution.

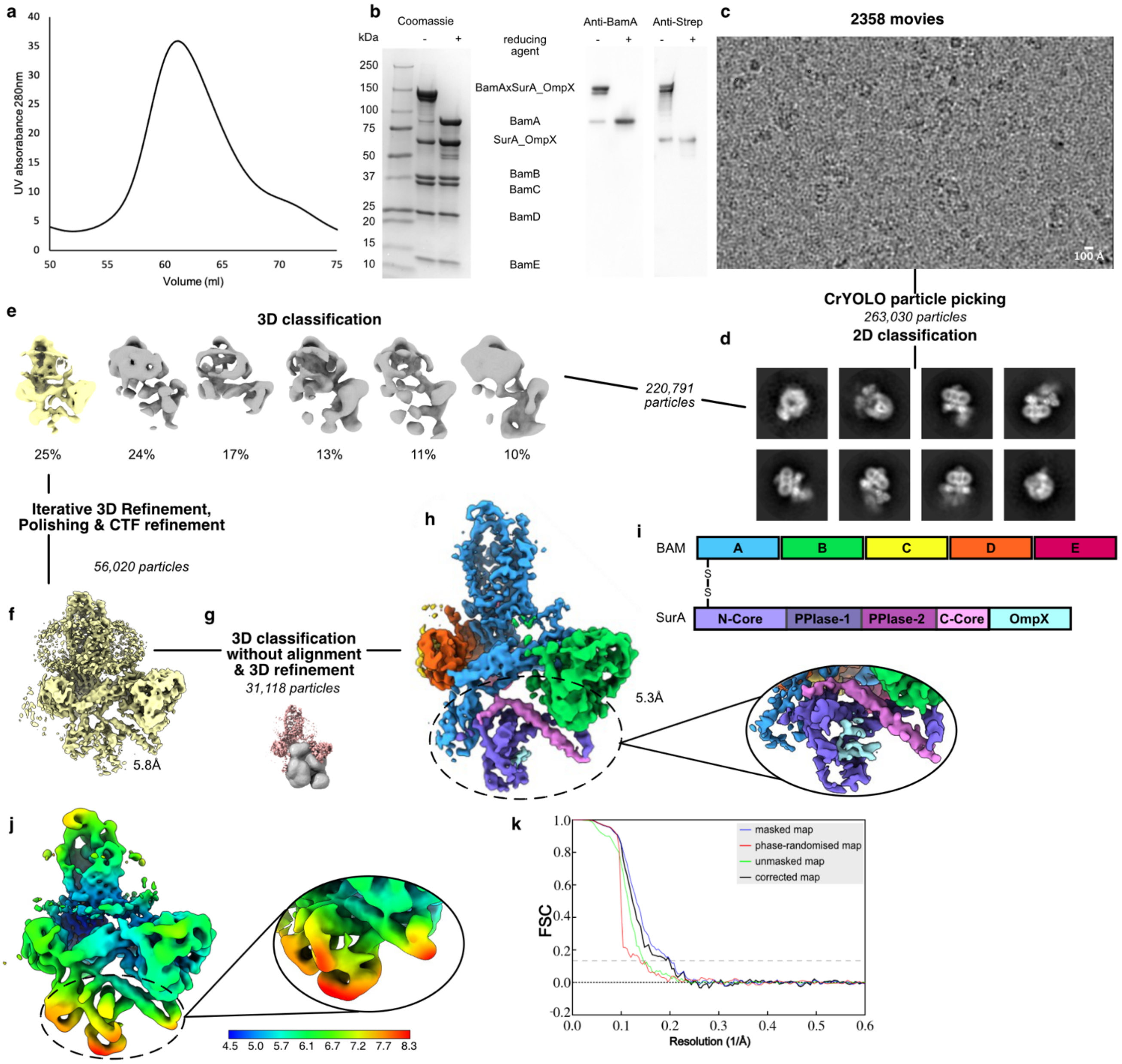

Supplementary Figure 7: CryoEM structure of SurA-OmpX:BAM cross-linked via POTRA-1.

a. Gel filtration trace for purification of BamA(R76C)BCDE:SurA(K27C)_OmpX. **b.** SDS PAGE gel and western blot of purified concentrated sample. SurA is Strep tagged. **c.** Representative raw electron micrograph and **d.** representative 2D classes. **e.** 6 class 3D classification, **f.** with 1 class with 25% of particles iteratively polished, CTF and 3D refined to a global resolution of 5.8 Å. **g.** 3D classification without alignment was used with the mask in grey to improve resolution of SurA and most abundant class consisting of 31,118 particles was 3D refined. **h.** Final reconstruction coloured by subunit (BAM) and domain (SurA-OmpX) with an inset focused on OmpX density. **i.** Schematic showing the location of the introduced disulphide bond and construction of SurA-OmpX hybrid. **j.** **F**inal reconstruction coloured by local resolution with an inset focused on OmpX density and **k.** FSC curve. (Source data are provided in the Source Data file for a and b)

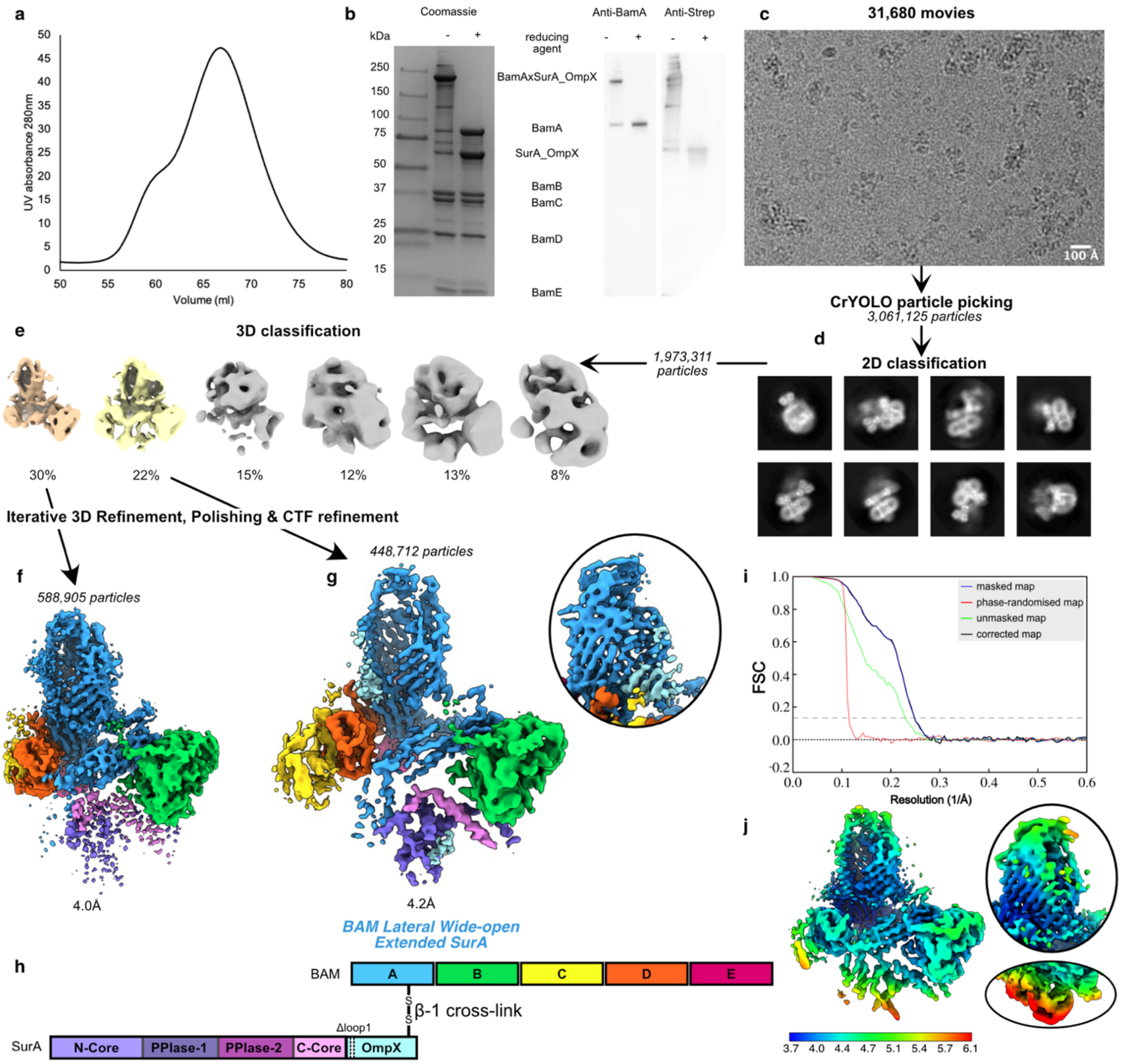

Supplementary Figure 8: CryoEM structure of SurA_OmpX cross-linked to β1 of BamA.

a. Gel filtration trace for purification of BamA(S425C)BCDE SurA_OmpX(R170C_Δloop1). **b.** SDS PAGE gel and western blot of purified concentrated sample (SurA is Strep tagged). **c.** Representative raw micrograph and **d.** representative 2D classes. **e.** 6 class 3D classification**. f.** Final reconstruction of BAM Lateral Open Extended SurA. **g.** Final reconstruction of BAM Lateral Wide-open Extended SurA with inset of wide barrel and three strands of OmpX. **h.** Final reconstructions coloured by protein subunit (BAM) and domain (SurA-OmpX). Schematic shows location of disulphide bond and construction of SurA-OmpX hybrid. **i.** FSC curve of BAM Lateral Wide-open Extended SurA and **j.** coloured by local resolution with insets of the OmpX density at the BamA barrel and in SurA Core. (Source data are provided in the Source Data file for a and b)

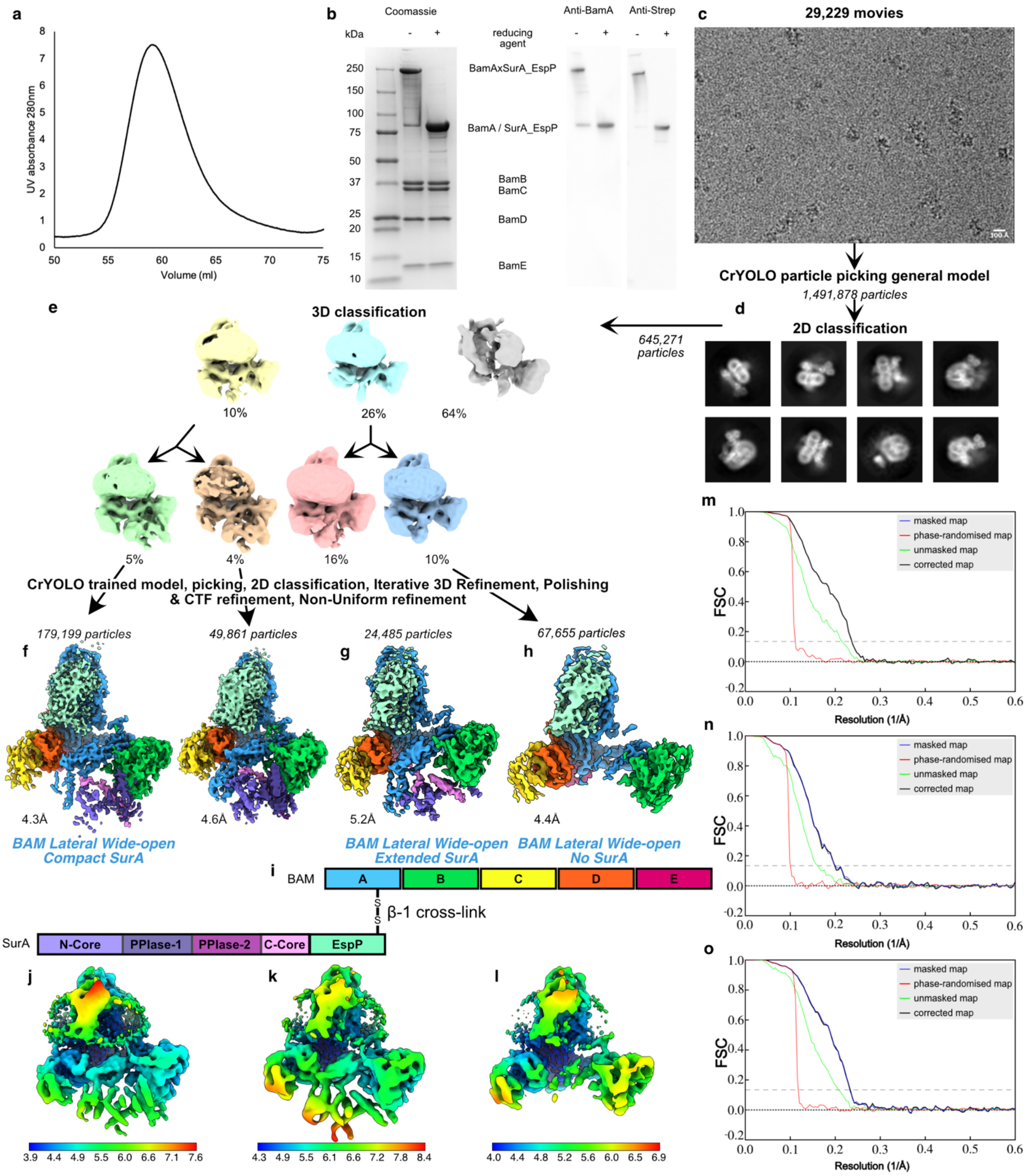

Supplementary Figure 9: CryoEM structure of SurA_EspP:BAM cross-linked between β1 of BamA.
a. Gel filtration trace for purification of BamA(S425C)BCDE SurA_EspP(S1299C). **b.** SDS PAGE gel and western blot of purified concentrated sample (SurA is Strep tagged). **c**. Representative raw micrograph and **d.** representative 2D classes. **e.** 3 class 3D classification with two classes further classified into two classes. Each class was used to trained a crYOLO model, repicked, 2D classified and iteratively 3D refined, polished and CTF refined before one round of non-uniform refinement in Cryosparc. Final reconstruction of **f.** BAM Lateral Wide-open Compact SurA, **g.** BAM Lateral Wide-open Extended SurA and **h.** BAM Lateral Wide-open No SurA. **i.** Final reconstructions coloured by protein subunit (BAM) and domain (SurA-EspP). Schematic show location of disulphide bond and construction of SurA-EspP hybrid. Maps coloured by local resolution of **j.** BAM Lateral Wide Open Compact SurA, **k.** BAM Lateral Wide-open Extended SurA and **l.** BAM Lateral Wide-open No SurA. FSC curves of **m.** BAM Lateral Wide-open Compact SurA, **n.** BAM Lateral Wide-open Extended SurA and **o.** BAM Lateral Wide-open No SurA. (Source data are provided in the Source Data file for a and b)

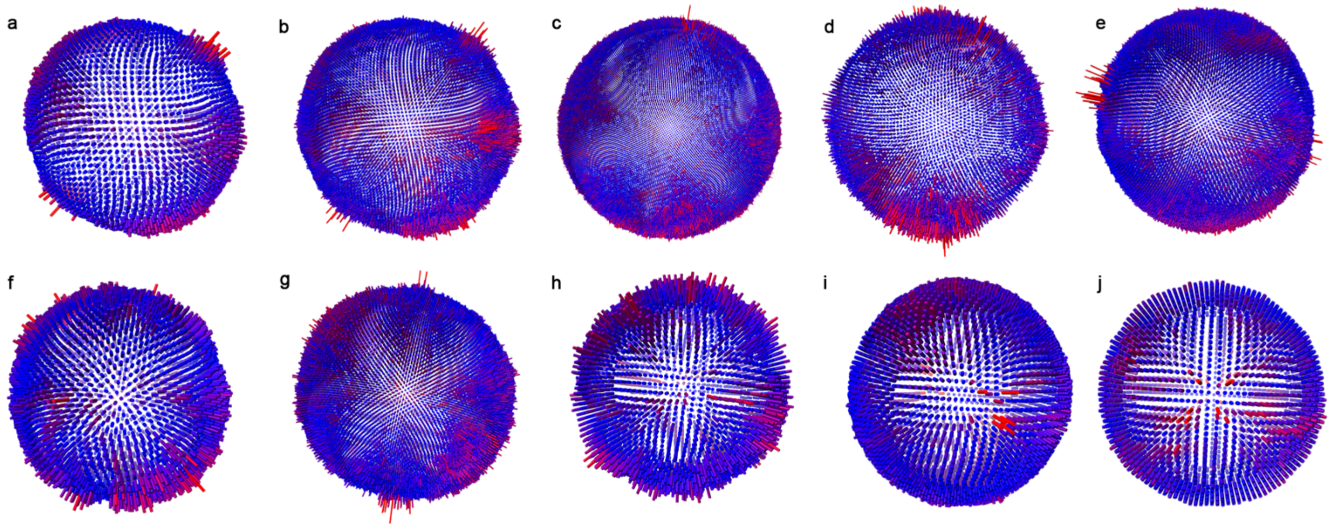

Supplementary Figure 10: Angular Distributions of final cryoEM maps.

Angular distributions of **a.** BAM lateral open Extended SurA and **b.** BAM lateral open Compact SurA from POTRA-1 crosslink dataset, **c.** BAM lateral closed Extended SurA and **d.** BAM lateral closed Compact SurA from POTRA-1 crosslink plus darobactin dataset **e.** BAM lateral open Extended SurA from POTRA-1 crosslink plus WEYIPNV dataset **f.** BAM lateral open Extended SurA with OmpX (Arrival complex) from POTRA-1 crosslink with SurA_OmpX **g.** BAM lateral wide open Extended SurA with OmpX (Handover complex) from β-1 crosslink with SurA_OmpX dataset **h.** BAM lateral wide open with EspP Extended SurA **i.** BAM lateral wide open with EspP Compact SurA **j.** BAM lateral wide open with EspP No surA from β-1 crosslink with SurA_EspP dataset

Supplementary Table 1: CryoEM data collection parameters.

Dataset	**POTRA1 cross-link**		**POTRA1 cross-link + darobactin B**	**POTRA1 cross-link + WEYIPNV**	**POTRA1 cross-link with SurA_ OmpX hybrid**	**β1 cross-link with SurA_ OmpX hybrid**	**β1 cross-link with SurA_EspP hybrid**
Microscope	Krios 1	Krios 1	Krios 1	Krios 2	Krios 2	Krios 1	Krios 1
Camera	Falcon4 Selectris	Falcon4 Selectris	Falcon4 Selectris	Falcon 4i	Falcon 4i	Falcon 4i Selectris	Falcon 4i Selectris
Magnification	165k	165k	130k	96k	96k	165k	165k
Pixel size (Å)	0.71	0.71	0.91	0.83	0.83	0.74	0.74
Micrographs	2,238	13,755	6,357	5,502	2,358	31,680	29,229
Dose per pixel/second	7.05	4.79	6.33	5.99	6.36	7.33	9.31
Dose per Å2/second	9.53	9.5	7.6	8.1	8.6	13.4	17
Exposure time	3.98	4.19	5.21	4.92	4.04	3.1	2.2
Total dose	37.9	39.8	39.8	39.8	34.7	41.5	37.4
Dose per frame	0.8	0.8	0.8	0.8	0.85	0.8	0.8
Frames	47	50	50	50	41	52	47

Supplementary Table 2: Modelling Statistics Table.

**Dataset**	**POTRA1 Cross-link**		**POTRA1 Cross-link + darobactin B**		**POTRA1 Cross-link OmpX Hybrid**	**β1 Cross-link OmpX Hybrid**	**β1 Cross-link EspP Hybrid**		
**Structure**	BAM Lateral Open<br>Extended SurA	BAM Lateral Open<br>Compact SurA	BAM Lateral Closed<br>Extended SurA	BAM Lateral Closed<br>Compact SurA	BAM Lateral Open<br>Extended SurA with OmpX	BAM Lateral Wide-open with OmpX<br>Extended SurA with OmpX	BAM Lateral Wide-open with EspP<br>Extended SurA	BAM Lateral Wide-open with EspP<br>Compact SurA	BAM Lateral Wide-open with EspP<br>SurA released
**EMDB Code**	EMD-18035	EMD-18034	EMD-18046	EMD-18045	EMD-18564	EMD-18563	EMD-18543	EMD-18053	EMD-18562
**PDB Code**	8PZ2	8PZ1	8PZV	8PZU	8QPW	8QPV	8QP5	8Q0G	8QPU
**EMPIAR**	EMPIAR-12197		EMPIAR-11933		EMPIAR-11939	EMPIAR-11940	EMPIAR-11941		
**Map**									
Pixel size (Å)	0.71	0.71	0.91	0.91	0.83	0.74	0.74	0.74	0.74
Resolution (Å) (0.143 FSC threshold)	4.2	4.1	2.9	3.5	5.3	4.0	5.2	4.3	4.4
Sharpening *B* factor (Å^2)	-156	-161	-82	-61	-215	-175	-217	-190	-178
**Model Refinement**									
Initial model (PDB)									
BAM	5LJO	5LJO	7NRI	8PZV	8PZ2	8PZ2	8PZ2	8PZ1	8QOG
SurA	1M5Y	1M5Y	8PZ2	8PZ1					
Substrate/inhibitor	-	-	7P1C	7P1C	Manually built	Manually Built	7TTC/3SLT	7TTC/3SLT	
Model resolution (Å) (0.5 FSC threshold)	4.0	4.0	2.9	3.5	4.7	4.0	4.6	4.2	4.2
Model map correlation	0.83	0.8	0.77	0.79	0.75	0.82	0.72	0.76	0.80
Model composition									
Non-hydrogen atoms	13124	13844	13307	13783	13300	14088	15060	15817	12548
Protein residues	1678	1776	1696	1764	1703	1825	2059	2166	1745
Ligands	0	0	2	2	0	0	0	0	0
Protein B factors (Å^2)	55.15	55.44	36.73	49.41	208.15	70.99	155.20	109.62	157.36
R.M.S. deviations									
Bond lengths (Å)	0.006	0.002	0.004	0.003	0.003	0.003	0.003	0.005	0.003
Bond angles (°)	0.696	0.54	0.635	0.631	0.631	0.654	0.641	0.690	0.608
Validation									
MolProbity score	2.09	1.88	2.02	1.94	2.15	1.96	2.15	2.18	2.15
Clashscore	11.53	9.39	11.46	12.97	15.35	10.32	15.38	14.89	14.68
Poor rotamers (%)	0.07	0	0	0.07	0.07	0.34	0.00	0	0.17
Ramachandran plot									
Favoured (%)	91.1	94.31	93.15	95.51	92.83	93.54	92.62	91.58	92.28
Allowed (%)	8.9	5.63	6.79	4.49	7.11	6.35	7.28	8.37	7.72
Disallowed (%)	0	0.06	0.06	0	0.06	0.11	0.10	0.05	0.00

Supplementary Table 3: List of Plasmids.

Plasmid name	Usage	Source
pTrc99a2-BamABCDE-CT8His	Expression	Roman-Hernandez et al. 2014
pTrc99a2-BamA(V75C)BCDE-CT8His	Expression	This study
pTrc99a2-BamA(R76C)BCDE-CT8His	Expression	This study
pTrc99a2-BamA(V77C)BCDE-CT8His	Expression	This study
pTrc99a2-BamA(V78C)BCDE-CT8His	Expression	This study
pTrc99a2-BamA(R79C)BCDE-CT8His	Expression	This study
pTrc99a2-BamA(D80C)BCDE-CT8His	Expression	This study
pTrc99a2-BamA(S425C)BCDE-CT8His	Expression	This study
pSCRhaB2-PelB-NTTwinStrep-TEV-SurA	Expression	This study
pSCRhaB2-SurA(P22C)	Expression	This study
pSCRhaB2-SurA(Q23C)	Expression	This study
pSCRhaB2-SurA(V24C)	Expression	This study
pSCRhaB2-SurA(V25C)	Expression	This study
pSCRhaB2-SurA(D26C)	Expression	This study
pSCRhaB2-SurA(K27C)	Expression	This study
pSCRhaB2-SurA(V28C)	Expression	This study
pET28a-PelB-NTTwinStrep-TEV-SurA_OmpX	Expression	This study
pET28a-SurA(K27C)_OmpX_loop1del-R170C	Expression	This study
pET28a-SurA(K27C)_EspP_S1299C	Expression	This study
pET28b-SurA$_{21\text{-}428}$ NT$_{6His}$	Expression	Schiffrin et al. 2020
pET28b-SurA$_{21\text{-}428}$(CysVariant)NT$_{6His}$	Expression	Schiffrin et al. 2020
pZA31-SurA	Complementation	This study
pZA31-SurA(ΔQ23-V28)	Complementation	This study
pZA31-SurA(Q23P)	Complementation	This study
pZA31-SurA(V24P)	Complementation	This study
pZA31-SurA(V25P)	Complementation	This study
pZA31-SurA(D26P)	Complementation	This study
pZA31-SurA(D26A)	Complementation	This study
pZA31-SurA(K27P)	Complementation	This study
pZA31-SurA(V28P)	Complementation	This study
pZS21-BamA	Complementation	Kim et al. 2007
pZS21-BamA(V75P)	Complementation	This study
pZS21-BamA(R76P)	Complementation	This study
pZS21-BamA(R76A)	Complementation	This study
pZS21-BamA(V77P)	Complementation	This study
pZS21-BamA(L78P)	Complementation	This study
pZS21-BamA(R79P)	Complementation	This study
pZS21-BamA(D80P)	Complementation	This study

Supplementary Table 4: List of primers.

Primer Description	Forward Sequence	Reverse Sequence
pTrc99a2-BamABCDE-CT8His mutation to BamA(V75C)	CTTTGAGGATTGCCGCGTCCTTCG	TTGCCGGTAGCAAACAGA
pTrc99a2-BamABCDE-CT8His mutation to BamA(R76C)	TGAGGATGTTTGCGTCCTTCGTG	AAGTTGCCGGTAGCAAAC
pTrc99a2-BamABCDE-CT8His mutation to BamA(V77C)	GGATGTTCGCTGCCTTCGTGATG	TCAAAGTTGCCGGTAGCA
pTrc99a2-BamABCDE-CT8His mutation to BamA(V78C)	TGTTCGCGTCTGCCGTGATGGTG	TCCTCAAAGTTGCCGGTA
pTrc99a2-BamABCDE-CT8His mutation to BamA(R79C)	TCGCGTCCTTTGCGATGGTGATAC	ACATCCTCAAAGTTGCCG
pTrc99a2-BamABCDE-CT8His mutation to BamA(D80C)	CGTCCTTCGTTGCGGTGATACCC	CGAACATCCTCAAAGTTG
pTrc99a2-BamABCDE-CT8His mutation to BamA(S425C)	CAACACCGGTTGCTTCAACTTTG	CGCTCTTTTACCTTGTAGAC
Insertion of PelB-NTTwinStrep-TEV-SurA into pSCRhaB2	TCTTCTCTCATCCGCCAAAACAGCCAAGCTGGGATTTAGTTGCTCAGG	GACTGGTCGTAATGAAATTCAGCAGGATCACATATGAAATACCTGCTGCCG
pSCRhaB2-PelB-NTTwinStrep-TEV-SurA mutation to SurA(P22C)	TCAGGGAGCCTGCCAGGTAGTCG	AAGTACAGGTTTTCGCCG
pSCRhaB2-PelB-NTTwinStrep-TEV-SurA mutation to SurA(Q23C)	GGGAGCCCCCTGCGTAGTCGATAAAG	TGAAAGTACAGGTTTTCG
pSCRhaB2-PelB-NTTwinStrep-TEV-SurA mutation to SurA(V24C)	AGCCCCCCAGTGCGTCGATAAAGTC	CCCTGAAAGTACAGGTTTTC
pSCRhaB2-PelB-NTTwinStrep-TEV-SurA mutation to SurA(V22C)	CCCCCAGGTATGCGATAAAGTCG	GCTCCCTGAAAGTACAGG
pSCRhaB2-PelB-NTTwinStrep-TEV-SurA mutation to SurA(D26C)	CCAGGTAGTCTGCAAAGTCGCAGCC	GGGGCTCCCTGAAAGTAC
pSCRhaB2-PelB-NTTwinStrep-TEV-SurA mutation to SurA(K27C)	GGTAGTCGATTGCGTCGCAGCCGTCG	TGGGGGGCTCCCTGAAAG
pSCRhaB2-PelB-NTTwinStrep-TEV-SurA mutation to SurA(V28C)	AGTCGATAAATGCGCAGCCGTCGTCAATAACGGC	ACCTGGGGGGCTCCCTGA
Insertion of OmpX into pSCRhaB2-PelB-NTTwinStrep-TEV-SurA	TAAGCACCATGGGCGACTTCTACTGTAACTGGC	TAAGCACTCGAGGAAGCGGTAACCAACACCG
Insertion of PelB-NTTwinStrep-TEV-SurA(K27C)-OmpX into pET28a	TAAGCAAAGCTTTGAGATCCGGCTGCTAACAA	TAAGCACATATGGGTATATCTCCTTCTTAAAGTTAAACAAAA
Creation of golden gate drop in vector pET28a-PelB-NTTS-TEV-SurA(K27C)_goldengate	CACATGGGTCTCCAAAGCTTTGAGATCCGGC	CACATGGGTCTCCCACCGTTGCTCAGGATTTTAACGT
pZA31-SurA deletion to SurA(Δ23-28)	GCAGCCGTCGTCAATAACG	GGGGGCAGCGAAACTGGT
pZA31-SurA mutation to SurA(Q23P)	CGCTGCCCCCCCGGTAGTCGATA	AAACTGGTATTCGCGATCATGGC
pZA31-SurA mutation to SurA(V24P)	TGCCCCCCAGCCGGTCGATAAAGTC	GCGAAACTGGTATTCGCG
pZA31-SurA mutation to SurA(V25P)	CCCCCAGGTACCGGATAAAGTCGCAGC	GCAGCGAAACTGGTATTC
pZA31-SurA mutation to SurA(D26P)	CCAGGTAGTCCCGAAAGTCGCAGCCG	GGGGCAGCGAAACTGGTA
pZA31-SurA mutation to SurA(D26A)	CCAGGTAGTCGCGAAAGTCGCAGC	GGGGCAGCGAAACTGGTA
pZA31-SurA mutation to SurA(K27P)	GGTAGTCGATCCGGTCGCAGCCGTCG	TGGGGGGCAGCGAAACTG
pZA31-SurA mutation to SurA(V28P)	AGTCGATAAACCGGCAGCCGTCGTCAATAACGGCGTCG	ACCTGGGGGGCAGCGAAA
pZS21-BamA mutation to BamA(V75P)	CTTTGAGGATCCGCGCGTCCTTCG	TTGCCGGTAGCAAACAGA
pZS21-BamA mutation to BamA(R76P)	TGAGGATGTTCCGGTCCTTCGTGATG	AAGTTGCCGGTAGCAAAC
pZS21-BamA mutation to BamA(R76A)	CACGAAGGACCGCAACATCCTCAAAGTTG	ATGGTGATACCCTTCTGG
pZS21-BamA mutation to BamA(V77P)	GGATGTTCGCCCGCTTCGTGATGGTG	TCAAAGTTGCCGGTAGCA
pZS21-BamA mutation to BamA(L78P)	TGTTCGCGTCCCGCGTGATGGTG	TCCTCAAAGTTGCCGGTAG
pZS21-BamA mutation to BamA(R79P)	TCGCGTCCTTCCGGATGGTGATAC	ACATCCTCAAAGTTGCCG
pZS21-BamA mutation to BamA(D80P)	CGTCCTTCGTCCGGGTGATACCC	CGAACATCCTCAAAGTTG

Supplementary Table 5: List of *E. coli* strains.

Strain name	Genotype	Source
BL21(DE3)	F^{-}*omp*T *hsd*S_B (r_B^{-}, m_B^{-}) *gal dcm* (DE3)	NEB
Lemo21(DE3)	fhuA2 [lon] ompT gal (λ DE3) [dcm] ∆hsdS/ pLemo(CamR) λ DE3 = λ sBamHIo ∆EcoRI-B int::(lacI::PlacUV5::T7 gene1) i21 ∆nin5 pLemo = pACYC184-PrhaBAD-lysY	NEB
JCM166	MC4100 ara^r Δ*bamA* Δ(*λatt-lom*)::*bla* P_{BAD}*bamA araC* Ap^r	Gift from Tom Silhavy[10]
AR208	MC4100 *surA*::kan	Gift from Tom Silhavy[11]

Supplementary Table 6: Single-molecule FRET PDA fitting results for SurA Core:PPIase1.

	SurA	SurA + BAM	SurA + WEYIPNV	SurA + BAM + WEYIPNV
**A_1**	0.188	0.508	0.671	0.768
**R_1 (Å)**	65.27			
**δ_1 (Å)**	5.70			
**A_2**	0.71	0.438	0.23	0.193
**R_2 (Å)**	54.52			
**δ_2 (Å)**	4.76			
**A_3**	0.04	0.018	0.017	0
**R_3 (Å)**	43.05			
**δ_3 (Å)**	3.76			
**A_4**	0.063	0.036	0.082	0.039
**R_4 (Å)**	10534.16*			
**δ_4 (Å)**	919.45*			
**Reduced χ^2**	4.01	2.36	1.93	1.82

Supplementary Table 7: Single-molecule FRET PDA fitting results for SurA Core:PPIase2.

	SurA	SurA + BAM	SurA + WEYIPNV	SurA + BAM + WEYIPNV
**A_1**	0.945	0.888	0.949	0.924
**R_1 (Å)**	64.36			
**δ_1 (Å)**	6.82			
**A_2**	0.042	0.063	0.003	0.047
**R_2 (Å)**	47.08			
**δ_2 (Å)**	4.99			
**A_3**	0.013	0.05	0.048	0.029
**R_3 (Å)**	102.77*			
**δ_3 (Å)**	10.89*			
**Reduced χ^2**	1.47	1.90	1.97	2.41

Supplementary References

1. Iadanza, M. G. *et al.* Lateral opening in the intact β-barrel assembly machinery captured by cryo-EM. *Nat. Commun.* **7**, 12865 (2016).
2. Haysom, S. F. *et al.* Darobactin B Stabilises a Lateral-Closed Conformation of the BAM Complex in E. coli Cells. *Angew. Chem.* **62**, e202218783 (2023).
3. Doyle, M. T. & Bernstein, H. D. Bacterial outer membrane proteins assemble via asymmetric interactions with the BamA β-barrel. *Nat. Commun.* **10**, 1–13 (2019).
4. Shen, C. *et al.* Structural basis of BAM-mediated outer membrane β-barrel protein assembly. *Nature* **617**, 185-193 (2023).
5. Bitto, E. & McKay, D. B. Crystallographic Structure of SurA, a Molecular Chaperone that Facilitates Folding of Outer Membrane Porins. *Structure* **10**, 1489–1498 (2002).
6. Xu, X., Wang, S., Hu, Y.-X. & Mckay, D. B. The Periplasmic Bacterial Molecular Chaperone SurA Adapts its Structure to Bind Peptides in Different Conformations to Assert a Sequence Preference for Aromatic Residues. *J. Mol. Biol.* **373,** 367-381 (2007).
7. Roman-Hernandez, G., Peterson, J. H. & Bernstein, H. D. Reconstitution of bacterial autotransporter assembly using purified components. *Elife* **3**, e04234 (2014).
8. Schiffrin, B. *et al.* Dynamic interplay between the periplasmic chaperone SurA and the BAM complex in outer membrane protein folding. *Commun. Biol.* **5**, 560 (2022).
9. Kim, S. *et al.* Structure and function of an essential component of the outer membrane protein assembly machine. *Science* **317**, 961–964 (2007).
10. Wu, T. *et al.* Identification of a Multicomponent Complex Required for Outer Membrane Biogenesis in Escherichia coli. *Cell* **121**, 235–245 (2005).
11. Rizzitello, A. E., Harper, J. R. & Silhavy, T. J. Genetic Evidence for Parallel Pathways of Chaperone Activity in the Periplasm of Escherichia coli. *J. Bacteriol.* **183**, 6794 (2001).
